# Modulation of attention networks serving reorientation in healthy aging

**DOI:** 10.18632/aging.103515

**Published:** 2020-06-24

**Authors:** Yasra Arif, Rachel K. Spooner, Alex I. Wiesman, Christine M. Embury, Amy L. Proskovec, Tony W. Wilson

**Affiliations:** 1 Department of Neurological Sciences, University of Nebraska Medical Center, Omaha, NE 68198, USA; 2 Center for Magnetoencephalography, University of Nebraska Medical Center, Omaha, NE 68198, USA; 3 Cognitive Neuroscience of Development and Aging (CoNDA) Center, University of Nebraska Medical Center, Omaha, NE 68198, USA; 4 Department of Psychology, University of Nebraska, Omaha, NE 68198, USA

**Keywords:** magnetoencephalography, oscillations, CRUNCH, validity effect, posner

## Abstract

Orienting attention to behaviorally relevant stimuli is essential for everyday functioning and mainly involves activity in the dorsal and ventral frontoparietal networks. Many studies have shown declines in the speed and accuracy of attentional reallocation with advancing age, but the underlying neural dynamics remain less understood. We investigated this age-related decline using magnetoencephalography (MEG) and a Posner task in 94 healthy adults (22-72 years old). MEG data were examined in the time-frequency domain, and significant oscillatory responses were imaged using a beamformer. We found that participants responded slower when attention reallocation was needed (i.e., the validity effect) and that this effect was positively correlated with age. We also found age-related validity effects on alpha activity in the left parietal and beta in the left frontal-eye fields from 350-950 ms. Overall, stronger alpha and beta responses were observed in younger participants during attention reallocation trials, but this pattern was reversed in the older participants. Interestingly, this alpha validity effect fully mediated the relationship between age and behavioral performance. In conclusion, older adults were slower in reorienting attention and exhibited age-related alterations in alpha and beta responses within parietal and frontal regions, which may reflect increased task demands depleting their compensatory resources.

## INTRODUCTION

Attentional orienting and re-orienting are critical to successful task performance across multiple cognitive domains and indispensable for daily functioning. Such attentional control is thought to be at least partially served by the activation of and interactions among frontoparietal regions of the two main attention networks, broadly classified as the dorsal and ventral attention networks (i.e., DAN and VAN) [1–15]. The DAN is comprised of the superior parietal lobules, intraparietal sulci, and frontal eye fields (FEF) and has been characterized as a top-down control mechanism (i.e., goal driven) [16–19], while the VAN is most often linked to the right temporoparietal junction (TPJ) and ventral frontal cortices and is thought to be more involved in the monitoring of bottom up processes (i.e., stimulus driven) [19].

Although certain subcomponents of attention processing have been shown to remain relatively unaffected by advancing age (e.g., spatial selective attention) [20–22], previous studies have demonstrated that aging has a major effect on attentional reorientation mechanisms in both the visual and auditory domains [23–25]. In fact, a large body of studies have shown age-related alterations in both the DAN and VAN, including reductions in gray matter volume primarily in the superior parietal [26], inferior parietal cortices [27, 28], and neuronal loss mainly in the magnocellular pathway [29–32]. Given the impact of age on the magnocellular pathway [33, 34], among the two attention networks, multiple studies have suggested that the DAN, specifically the superior parietal cortices and the inferior parietal/intraparietal sulci [27], is relatively more susceptible to the aging process than the VAN. This increased susceptibility is partially because its major input is from the lateral geniculate nucleus via magnocellular fibers, whereas the VAN receives input from both magnocellular and parvocellular pathways [35–38], although see [39] for contradictory findings. While extensive research on aging and attention has been conducted over the past few decades, the vast majority of work in this area has focused on brain structure or used functional MRI (fMRI), and thus the impact of aging on the oscillatory dynamics serving attentional orienting remains far less clear.

In the current study, we utilized magnetoencephalography (MEG) and a modified Posner task to probe the neural dynamics underlying attentional reorienting as a function of healthy aging in a large sample of 94 adults (age range 22-72 years). MEG provides high temporal resolution (i.e., millisecond scale) and good spatial precision (i.e., sub-centimeter), which enables multispectral responses to be distinguished and the component operations that contribute to attentional reorientation to be directly examined. In addition, the Posner task is well-established and has been used extensively to study attention shifts [40–43], including in a previous study from our laboratory that showed multispectral activity in healthy young adults across both the DAN and VAN during task performance [6]. During the Posner task, participants are generally presented with a cue preceding a target stimulus. Depending on its location, the cue can either be valid, such that it appears in the same location as that of the target, or invalid, such that it appears in a different location than that of the target. In the face of an invalid cue, participants are required to reorient their attention to the target [1]. The difference between validly and invalidly cued trials is generally referred to as the validity effect (i.e., invalid – valid) [14], and represents the cost of reorienting attention [1]. Participants typically respond more slowly to invalid versus valid trials and this behavioral validity effect is generally interpreted as the cost of reorienting attention. Based on previous literature, our hypotheses were that healthy aging would result in larger behavioral validity effects (i.e., slower attention reorientation) and altered alpha and beta neural oscillations across multiple nodes of the VAN and DAN. Further, we predicted that these neural alterations would be consistent with the compensation-related utilization of neural circuits hypothesis (i.e., CRUNCH) [44], which posits that, under lower cognitive demands, older adults maintain adequate performance by engaging greater volumes of brain tissue relative to younger adults, but that under higher demands older adults have already exhausted the capacity of these compensatory brain circuits resulting in poorer task performance. Lastly, we also hypothesized that neural oscillatory activity within attention nodes would predict the impact of aging on the reaction time validity effect.

## RESULTS

**Figure 1:**
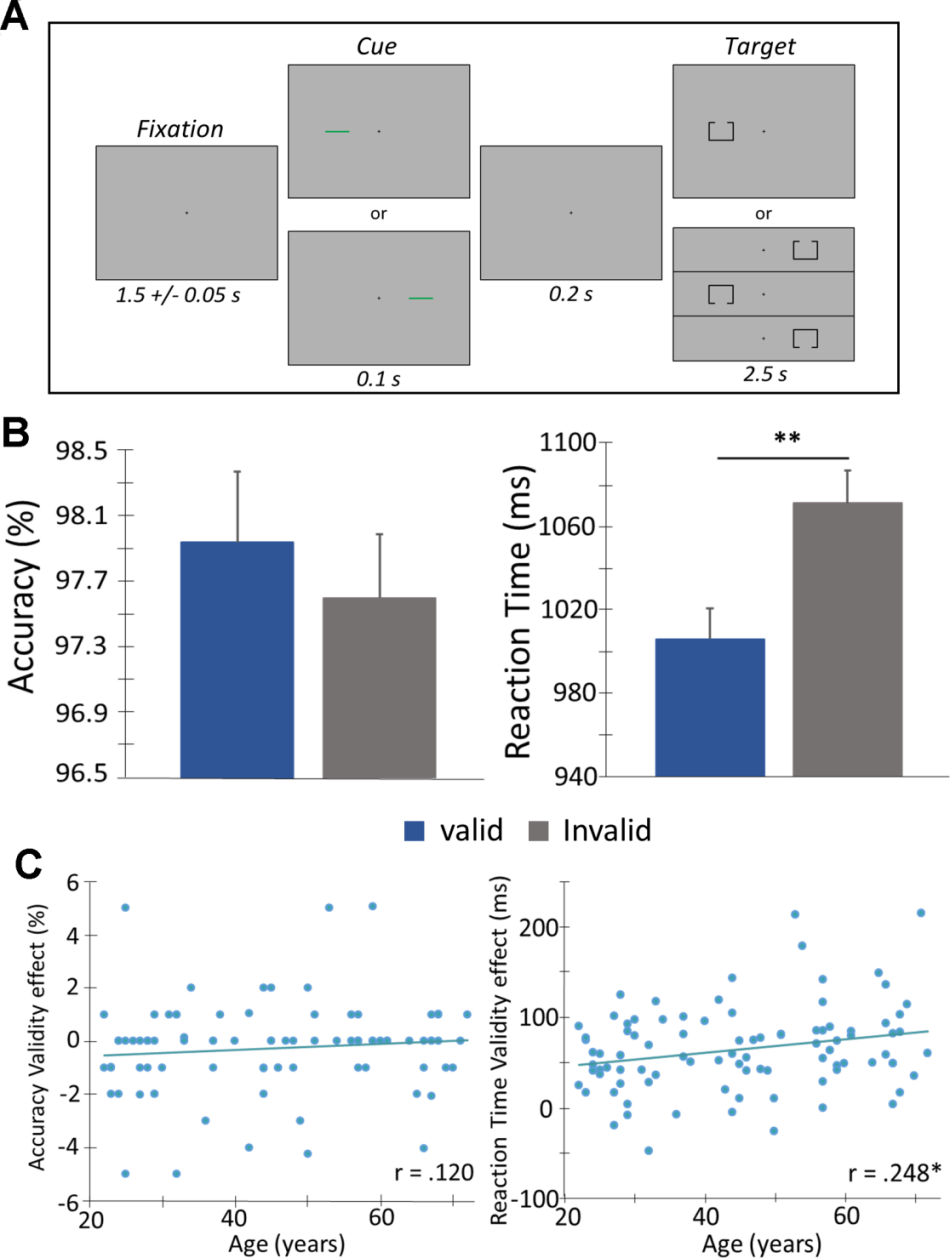
**Posner cueing task and behavioral performance.** (**A**) A central crosshair was presented for 1500 ms (± 50 ms), followed by a cue (green bar) that appeared in either the left or right hemifield for 100 ms. Target presentation (box with opening at the top or bottom) was presented 200 ms after cue offset, in either hemifield for 2500 ms. The cue was predictive of the upcoming target location 50% of the time (i.e., “valid” condition - presented on same side as the subsequent target). Participants completed 200 trials and were instructed to respond as to whether the opening was on the bottom (right index finger) or top (right middle finger) of the box. Trials were pseudorandomized and counterbalanced in regard to target validity (valid or invalid), visual hemifield (left or right), and box opening (top or bottom). (**B**) Behavioral metrics are displayed on the y-axis with conditions (valid or invalid) on the x-axis. Irrespective of age, participants were faster to respond and more accurate during valid compared to invalid trials. (**C**) Behavioral validity effect scores (invalid – valid) were computed for accuracy and reaction time and assessed as a function of age using Pearson correlations. There was a significant correlation among reaction time validity and age, such that as age increased, the difference in the reaction time between the two conditions (i.e., valid and invalid) increased. Error bars reflect the SEM. ** *p* < .05.

Ninety-four healthy adults (42 females, 14 left-handed) with a mean age of 45.60 years, SD = 15.6 (range: 22-72 years) successfully completed the Posner task (Figure 1A), but two were excluded due to poor behavioral performance (i.e., very delayed responses that were greater than 2.5 SD above the sample mean).

### Behavioral effects

Analysis of covariance on the behavioral data showed a significant interaction of condition by age for reaction time, (F(1,90) = 5.874, *p* = .017), such that the slower responses in the invalid relative to valid condition became greater with increased age (Figure 1B, 1C). However, no such interaction was found for accuracy, (F(1,90) = 1.314, *p* = .255). Simple effect tests showed a main effect of condition on reaction time, (t(91) = -14.15, *p* < 0.001), such that participants responded more slowly during invalid trials relative to valid trials (Figure 1B). This validity effect [14] was 66.93 ms on average (SD = 45.13). However, the difference in accuracy between the two conditions was marginal, (t(91) = 1.84, *p* = .069), and the performance was near ceiling in both conditions (valid trials (M = 97.96%, SD = 0.04, invalid trials (M = 97.62%, SD = 0.04).

### Sensor level analysis

**Figure 2:**
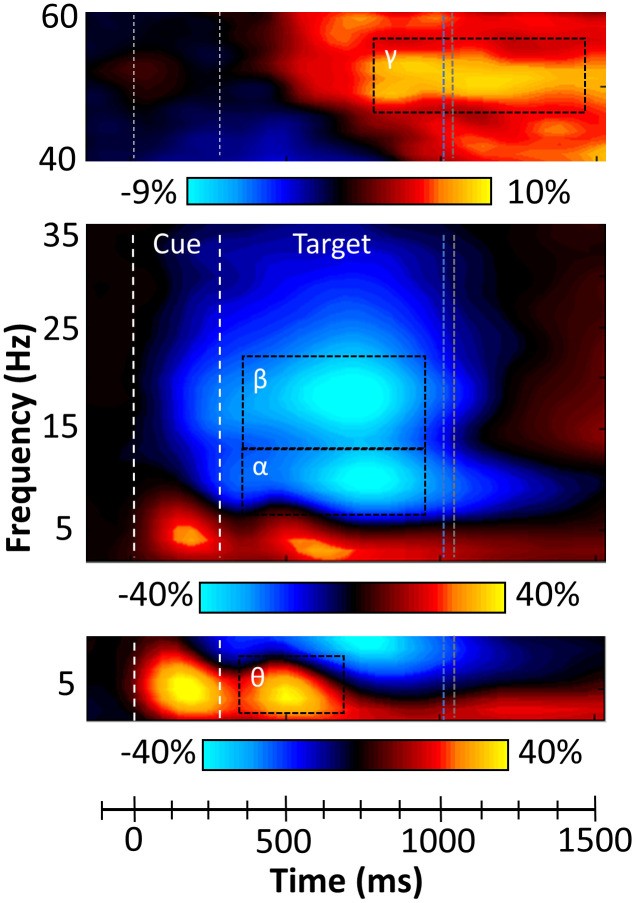
**Sensor level time-frequency analysis.** Grand averaged spectrograms for two sensors near parietal cortices with time (ms) displayed on the x-axis and frequency (Hz) denoted on the y-axis. Power is shown in percentage units relative to the baseline period (-600 to 0 ms), with a color scale bar beneath each spectrogram. The data per spectrogram have been averaged across all trials and participants. (Bottom) A strong increase in theta (3-7 Hz) power was observed following cue onset and during target processing (350-700 ms). (Middle) Strong decreases in alpha (8-14 Hz, 350-950 ms) and beta (14-22 Hz, 350-950 ms) power were also observed after the onset of the target. (Top) Robust increases in gamma (46-58 Hz) activity occurred during later target processing (850-1450 ms). All four oscillatory responses significantly differed from baseline activity in the same spectral band (*p* < .001, corrected), and these time-frequency windows have been highlighted using the black dotted line boundaries. Vertical blue and grey dotted lines represent the average reaction times for valid and invalid trials, respectively.

While strong theta and alpha/beta responses were observed after cue onset, the goal of the current study was to examine oscillations related to the attentional reorienting process. Thus, our statistical analyses focused on neural activity during the target period (i.e., starting 300 ms after cue onset). These analyses revealed four spectrally specific oscillatory responses in gradiometers near the parietal, occipital, and frontal cortices across all participants and both conditions (Figure 2). Briefly, during the target presentation, a strong increase in the theta range (3-7 Hz) was observed from 350 to 700 ms (*p* < .001, corrected). This response partially overlapped in time with robust decreases in the alpha (8-14 Hz; 350-950 ms, *p* < .001, corrected) and beta ranges (14-22 Hz; 350-950 ms, *p* < .001, corrected). Finally, a strong gamma increase (46-58 Hz; 850-1450 ms, *p* < .001, corrected) was observed and this oscillatory response was most prominent in sensors near the occipital cortices.

### Beamformer analysis

**Figure 3:**
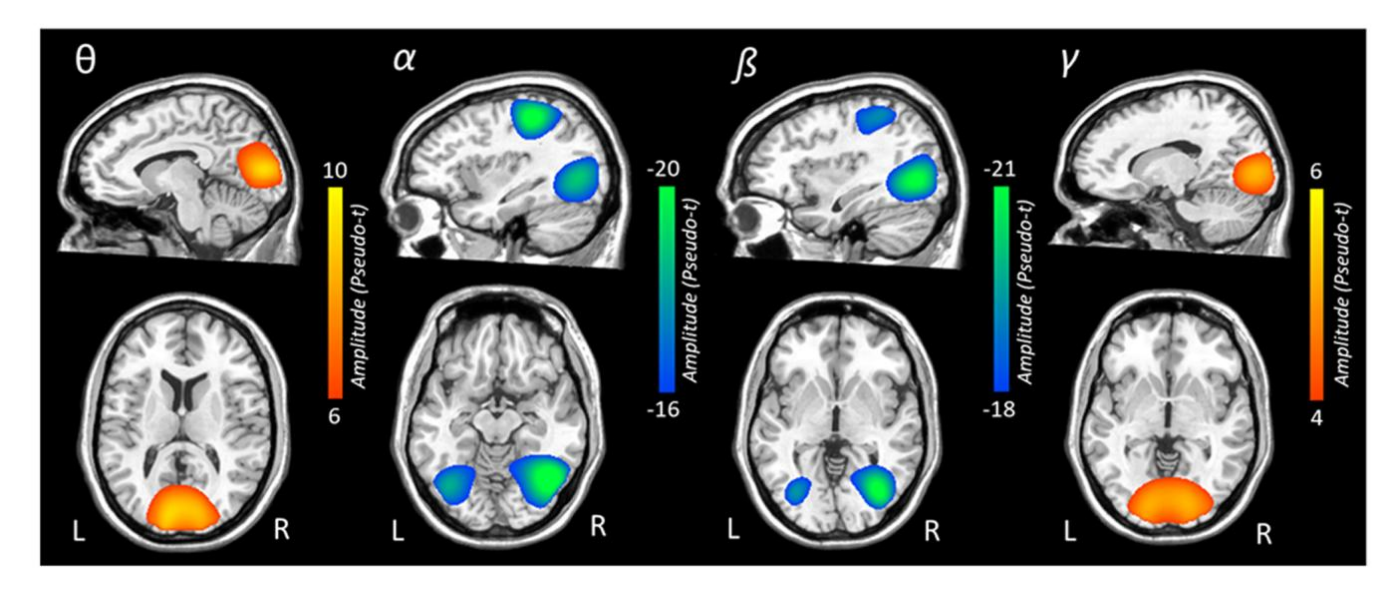
**Grand-averaged beamformer images (pseudo-t) for each oscillatory response.** In each image, data from both conditions and all participants have been grand averaged. Theta responses were strongest in bilateral medial occipital cortices. In contrast, robust decreases in alpha activity were seen in more lateral occipital cortices bilaterally and the left superior parietal lobule. Decreases in beta activity were also observed in bilateral occipital cortices and the left intraparietal sulcus. Gamma-frequency responses were strongest in the medial bilateral visual cortices.

To identify the spatial origin of these sensor level oscillatory responses, the aforementioned time-frequency windows of interest were imaged using a beamformer, and the resulting maps per response were averaged over both conditions and across all participants. Strong increases in theta activity were observed from 350-700 ms in bilateral occipital cortices, bilateral inferior frontal gyri, and the right cingulate gyrus. In contrast, strong decreases in alpha activity were observed in lateral occipital cortices bilaterally, left superior parietal lobule, and the left precuneus from 350-950 ms. Likewise, strong decreases in beta activity were observed in bilateral occipital cortices and the left intraparietal sulcus. Finally, the robust increase in gamma activity from 850-1450 ms originated in the bilateral occipital cortices (Figure 3). To illustrate the quality of the data and consistency of task-related neural responses with previous normative studies that used the same Posner task [6, 43], average maps across both conditions and all participants are shown per oscillatory response in Figure 3.

**Figure 4:**
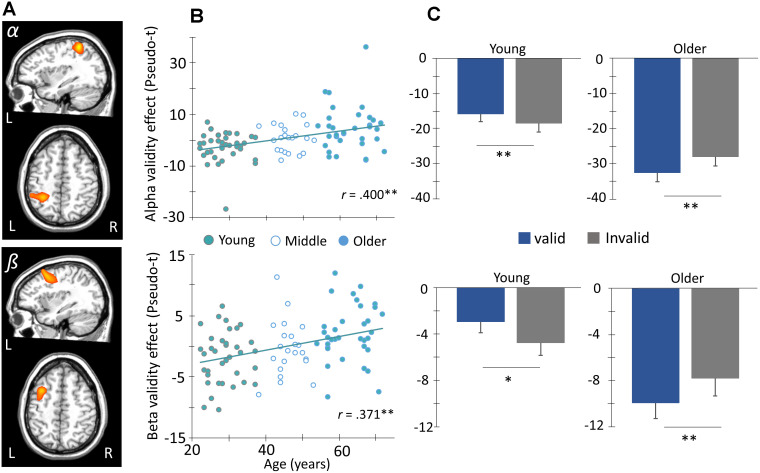
**Age modulates frontoparietal networks during attentional reorienting.** (**A**) Whole-brain voxel-wise correlational analysis of alpha (8-14 Hz, top) and beta (14-22 Hz, bottom) validity maps (i.e., invalid – valid) and age revealed significant positive correlations between alpha and beta validity effects in left parietal cortex and frontal eye fields (FEF), respectively, and age. Images in two planes are shown for each. (**B**) The amplitude (pseudo-t) of the peak voxels shown in (**A**) were extracted and plotted as a function of age (x-axis) to identify the origin and distribution of the age-validity effect. Again, the parietal alpha data appears on the top with the frontal beta below. (**C**) Given this finding, the sample was stratified into age groups (i.e., younger and older) based on ± 0.5 SD of the full group’s mean age, such that subjects above 0.5 SDs were defined as the older group, and those below 0.5 SDs were defined as the younger group. This stratification can be seen in (**B**). The average amplitude of each conditional response (valid and invalid) is plotted to the right. Post-hoc paired t-tests were then conducted to identify the direction of the validity effect in each group. Asterisks mark significant validity effects (*p* < .05), with error bars reflecting the SEM. ** *p* < .001.

To determine the impact of aging on oscillatory activity underlying attention reallocation, difference maps were computed by subtracting whole brain voxel-wise maps of validly cued trials from invalidly cued. The resulting “validity effect” maps (i.e., attention orienting trials – nonorienting trials) were then subjected to whole brain voxel-wise correlation analyses with chronological age, and the resultant significant clusters (*p* < 0.01) were subjected to nonparametric permutation testing to control for Type 1 error. The *p*-values reported below correspond to the results of the permutation tests. Our results indicated significant age-related validity effects for alpha activity (8-14 Hz, 350-950 ms; *p* = 0.039, corrected; Figure 4A) in the left superior parietal region extending inferior along the intraparietal sulcus, such that increasing age was associated with a smaller alpha validity effect in this region. Significant age-related effects were also observed for beta activity (14-22 Hz, 350-950 ms; *p* = 0.020, corrected; Figure 4A) in the left frontal eye fields, such that increasing age was again associated with a smaller or reverse validity effect. To better visualize the correlations, peak voxels were extracted from each cluster and plotted against age (Figure 4B). These scatterplots showed that the peak relationship between age and the neural validity effect in these regions was robust or both alpha (r = 0.40, *p* < 0.001) and beta (r = 0.371, *p* < 0.001). Note that these *p*-values only correspond to the peak- voxel and that the cluster-level statistics should be considered primary. Finally, to probe the source of these age-related validity effects, the sample was divided into older and younger groups. Briefly, participants who fell within ± 0.5 SDs of the full group’s mean age were excluded from this aspect of the analysis, while those above 0.5 SD became members of the older group, and those below 0.5 SD became members of the younger group. This yielded two groups with the following age characteristics (Young: N = 36, M = 28.58 years old, SD = 4.34; Old: N = 34, M = 63.03 years old, SD = 5.34), and post-hoc paired t-tests were used to test the conditional effect in each group. The results indicated significantly stronger alpha and beta (*p*s < 0.05) responses during invalid relative to valid trials in the younger group, whereas the opposite pattern was observed for both alpha and beta (*p*s < 0.05) in the older group (Figure 4C). Lastly, no age-related validity effects were observed for theta or gamma oscillatory activity following our stringent statistical testing, and exploratory analyses examining interactions with sex were all non-significant.

### Mediation analysis

**Figure 5:**
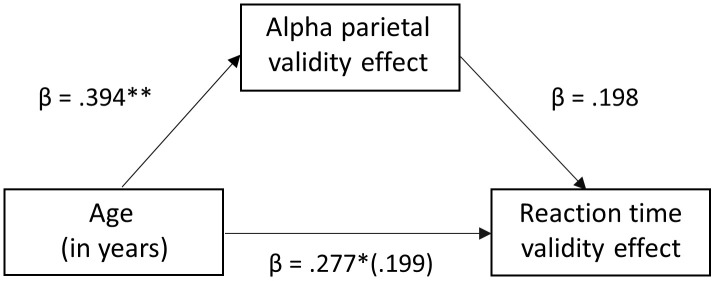
**Mediation analysis of age on reaction time validity through the mediator (neural validity).** There was a significant full mediation of age on reaction time validity through the mediator (i.e., alpha validity effect in the left parietal cortex), such that the increase in reaction time validity scores (i.e., cost of attention reallocation) with increased age was driven by stronger alpha desynchronizations to valid relative to invalidly-cued trials in the left parietal cortex. Each arrow is labeled with the standardized Beta coefficient values for the respective regression model. * *p* < .01, ** *p* <.005.

To determine the role of these oscillatory responses in parietal cortices and the frontal eye fields (FEF) in the larger reaction time validity effect with increasing age, a mediation analysis was conducted [45], followed by bootstrapping [46]. We hypothesized that the relationship between age and the reaction time validity effect (i.e., attention reallocation) would be mediated by the neural validity effect in these brain regions (i.e., alpha parietal, beta FEF). To analyze the indirect effects of the neural validity effect on the relationship between age and reaction time validity, three regression models were ran for each alpha and beta bands. The first was a simple regression of reaction time validity on age. It was found to be significant for both alpha (F(1, 89) = 7.309, *p* < .008, *R*^2^ = .077) and beta (F(1, 90) = 5.972, *p* < 0.017 *R*^2^ = .063). Following that, the simple regression models of neural validity on age were conducted and were also found to be significant for both alpha (F(1,89) = 16.141, *p* < 0.001, *R*^2^ = .155) and beta (F(1,90) = 14.682, *p* < 0.001, *R*^2^ = .376). Finally, multiple regression models of reaction time validity on age and neural validity were performed. The model of reaction time validity on age and alpha parietal validity was found to be significant (F(2, 89) = 5.361, *p* = 0.006, *R*^2^ = .11) indicating that together, age and alpha parietal validity were a significant positive predictor of reaction time validity. However, interestingly, the relationship between age and reaction time validity became non-significant after alpha parietal validity was added into the model, with the standardized regression coefficient decreasing from .277 to .199 (Figure 5). In contrast, the model of reaction time validity on age and beta FEF validity was not significant (F(2, 89) = 4.671, *p* = 0.114, *R*^2^ = .31). Moreover, after addition of beta FEF validity to the model, the relationship between age and reaction time validity remained significant, showing a direct relationship and no mediation (*p* = 0.004). For added rigor, bootstrapping was conducted and interestingly, our results confirmed full mediation of the age/RT-validity effect by the parietal alpha validity effect, *b* = 0.228, Z = 1.60, 95% CI (.0287, .4686; Figure 5), suggesting that alpha activity in the parietal cortex accounts for the age-related decline in behavioral performance.

## DISCUSSION

**Figure 6:**
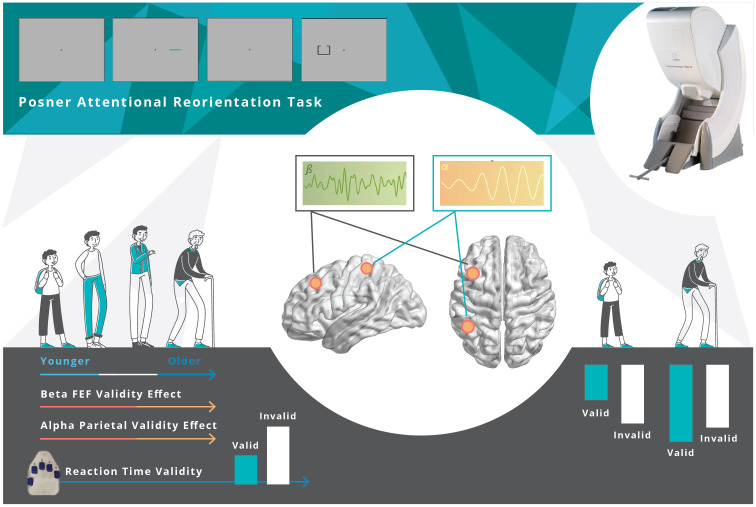
**Overall Findings.** Healthy aging modulates the behavioral and neural responses underlying attentional reorientation. The decrement in reaction time to invalid versus valid trials increased as a function of aging. Younger adults uniquely utilized parietal alpha and FEF beta activity, mainly in response to invalid trials, but this compensatory process became exhausted with increasing age.

Herein, we employed spatially resolved MEG and a well-known attention reorienting paradigm (i.e., the Posner cueing task) [40] to investigate the effects of healthy aging on the behavioral and oscillatory dynamics serving attentional reorienting. Our advanced oscillatory analyses enabled us to identify a complex pattern of spectrally specific neural responses serving the reallocation of attention, and further, these responses were significantly modulated by the aging process (Figure 6). Specifically, cue validity-related alpha and beta oscillations in regions of the DAN (i.e., left parietal and left FEF) were significantly correlated with age. Finally, left parietal neural validity effects in the alpha band fully mediated the effect of age on behavioral performance. These findings and their implications are discussed below in further detail.

Our behavioral findings supported our hypotheses and previous literature using the Posner task. Specifically, we observed increased response time on invalid relative to valid trials (i.e., validity effect), as well as increased reaction time validity effects as a function of age. Indeed, we observed that participants were slower to respond to invalid trials as compared to valid trials and that this decrement in reaction time was potentiated with increased age. These changes in reaction time are in broad accordance with the literature on increased processing time and deficient spatial orienting with aging [6, 47–50].

In regard to the neural findings, we observed robust validity effects in discrete spectral bands that were significantly modulated by healthy aging. For example, we observed that validity-related decreases in left parietal alpha activity decreased as a function of increasing age, such that the substantially stronger decrease in alpha amplitude during invalid relative to valid trials seen in younger adults was essentially abolished in their older counterparts. In other words, while older adults qualitatively exhibited more robust alpha oscillatory responses in the left parietal cortex compared to younger adults, the stronger responses during invalid relative to valid trials seen in young adults were no longer present. This same pattern of activity was observed for beta oscillations in the left FEF, with the greater discrepancy in power during invalid compared to valid trials, with increasing age. While alpha and beta synchronizations have been extensively linked to inhibition of active cognitive processing, alpha/beta desynchronizations in a region reflect the active engagement of that region during cognitive processing [51–54]. A large body of EEG/fMRI literature consolidates it further by reporting a negative correlation between BOLD signal and alpha/beta desynchronizations [55–58]. Ultimately, our data aligns nicely with previous reports that reduced alpha and beta power in left parietal and FEF regions signals increased engagement of these areas during target processing.

Notably, there have been numerous reports of frontoparietal networks involved in attentional processing, including the reorienting of attention. Constituting major portions of the DAN, both parietal cortex and FEF have been implicated in the top down control and selection of goal relevant stimuli and covert voluntary attentional engagement. For example, a fMRI study of target detection reported sustained activation within parietal cortices during voluntary attentional orienting [12], while another event related fMRI study showed active engagement of frontal and parietal regions in response to cue presentation; in both studies, the activation was interpreted as top down modulation of attention [59]. Additionally, another fMRI study focused on the neural correlates of reorienting attention and showed stronger responses in the frontal and parietal cortices during invalid as compared to valid trials while performing a cued target detection task [60]. Further support is provided by a recent EEG study whereby modulation of the alpha band in the parietal region was tied to inter-sensory re-orienting during the presentation of a trimodal stimulus set involving visual, auditory, and tactile stimuli [61]. Moreover, many transcranial magnetic stimulation (TMS) studies have also reported on the role of FEF and parietal cortex in orienting visuospatial attention such that TMS over FEF enhances visual detection and parietal stimulation facilitates voluntary orientation [62–67]. This framework has also been supported by several PET studies, which further show the activations of the above-mentioned regions during shifts of visuospatial attention [68, 69].

The parietal cortex has been shown to play a pivotal role in attentional reorienting in a large set of fMRI studies, such that stronger increases in functional activation follow the presentation of a spatially invalid cue [14, 60, 70]. Further, people with parietal lesions have been found to have difficulties in not only the redirection of attention but also in disengaging their attention from an expected target location to another in the face of invalid prompts [71, 72]. This disengagement of attention from spatial cues has also been shown to be aberrant in older age [73–75]. To compensate for this, broadly speaking, older people tend to have greater activation of frontoparietal regions during cognitive processing [45, 76, 77]. Altogether, previous reports align well with the current findings, which suggests that in older participants, there is a general increase in alpha desynchronization in the left parietal cortex during attentional processing, with stronger alpha responses (i.e., desynchronizations) during the processing of valid compared to invalid targets, a phenomenon that is essentially reversed in their younger counterparts. This reversed neural validity effect (i.e., greater activity during valid compared to invalid trials) in parietal regions may suggest an inefficiency in visuospatial disengagement in aging. Additionally, our mediation analysis suggested that the effect of age on reaction time validity was driven by the altered alpha validity effect in the left parietal cortex, which suggests that activity in the parietal cortices with aging is largely responsible for the behavioral decrement (i.e., slower to respond to invalid versus valid trials) in older adults.

Finally, findings from the current study hold important implications for beta activity in frontal regions during attentional reorienting in healthy aging. Specifically, we report increases in the beta validity effect as a function of age in the FEF. As with alpha activity in the parietal cortex, older participants showed generally stronger beta desynchronization responses regardless of condition compared to younger adults, as well as stronger oscillatory activity in response to valid trials compared to invalid. Neuronal activity in the FEF has been tightly linked by multiple studies to the covert allocation of visuospatial attention to behaviorally relevant stimuli [1, 3, 14, 78–80]. Studies targeting the FEF with micro-stimulation and/or TMS also provide evidence for the indispensable role of this region in orienting attention to relevant visual targets both in monkeys as well as in humans [65, 81, 82]. Moreover, unilateral lesions or pharmacological inactivation of the FEF in macaque monkeys has been shown to cause transient contralateral neglect [83–85]. Thus, our results further support the role of the FEF in attentional orientation and extend this notion across age groups. In the event of attentional reorienting, previous literature supports stronger frontal recruitment when participants had to reallocate their attention to a new location [6, 86–89]. However, our finding of a reversal of this pattern of activity in older adults likely reflects that, although the beta desynchronization in the left FEF was relatively stronger overall (i.e., across both conditions), older adults may have exhausted their resources as the cognitive demand increased with the need for attentional reallocation. In other words, they exhibited very strong responses during the more basic valid trials, and the need to redirect attention from an attended to unattended hemifield during the presentation of the invalid trials may have resulted in exceeding a resource capacity limit and ultimately decreased neural responses in the FEF [90–93]. Such an interpretation would be consistent with the compensation-related utilization of neural circuits hypothesis (i.e., CRUNCH) [44]. CRUNCH extends upon the typical age-related compensation hypothesis by clarifying that, under lower cognitive demands, older adults engage greater volumes of cortical tissue during task performance relative to younger adults, which aids in successful performance. However, under higher demands, older adults have already exhausted their compensatory circuits and reached a resource ceiling, resulting in poorer task performance. Meanwhile, younger adults are able to engage these compensatory circuits to meet the increased cognitive demands. Support for CRUNCH comes from neuroimaging studies that showed that age-related differences in PFC activation varied with task demands and are broadly consistent with the alpha and beta results in parietal cortices and the FEF, respectively, in the current study, as well as the overall pattern of behavioral results.

In conclusion, stronger recruitment of parietal and frontal regions in response to attentional reorienting in older adults, along with decreases in behavioral performance, agrees broadly with the CRUNCH hypothesis [45]. Additionally, as task demands increased (i.e., presentation of invalid trials), older adults appear to have already depleted their compensatory mechanisms and reached a resource ceiling, leading to poorer task performance (i.e., increased reaction time to invalid as compared to valid trials). Importantly, our study adds significant new data concerning the oscillatory dynamics of these age-related alterations and should be especially robust, given the large sample size and rigorous statistical approach used herein. Future studies should evaluate how these dynamics are affected in pathological conditions associated with aging, such as Alzheimer’s disease, hypertension, diabetes, and HIV.

## MATERIALS AND METHODS

### Participants

Ninety-four adults (42 females, 14 left-handed) with a mean age of 45.60 years, SD = 15.6 (range: 22-72 years) were enrolled in this study. Exclusionary criteria included any medical illness affecting CNS function (e.g., HIV/AIDS, lupus), cognitive impairment based on neuro-psychological testing, any neurological or psychiatric disorder, history of head trauma, current substance abuse, and the MEG laboratory’s standard exclusion criteria (e.g., ferromagnetic implants). Written informed consent was obtained from each participant after a thorough description of the study was provided, following the guidelines of the University of Nebraska Medical Center’s Institutional Review Board, which approved the study protocol.

### Experimental paradigm

The paradigm used in this study was a modified Posner task (Figure 1A) [40]. During this task, the participants were seated in a magnetically shielded room and told to fixate on a crosshair presented centrally for 1500 ms (± 50 ms). Following that, a green bar (the cue), was presented either to the left or right of the crosshair for 100 ms. The cue appeared on a given side 50% of all trials and could either be valid (presented on the same side as the upcoming target, 50% of all trials) or invalid (presented on the opposite side relative to the target). At 300 ms (200 ms after cue offset), a target was presented on either the left or the right side of the crosshair for 2500 ms, and this was comprised of a box with an opening on either its top (50% of trials) or bottom. Participants were instructed to respond as to whether the opening was on the top (right middle finger) or the bottom (right index finger) of the box. Trials where the participant responded more than 2.5 SD beyond the mean reaction time were excluded. Each target variant appeared an equal number of times and each trial lasted 4300 ms (± 50 ms). A total of 200 trials were used (100 valid, 100 invalid), leading to a total run-time of approximately 14.5 minutes. Trials were pseudo-randomly organized so that no more than three of the same target response or target/cue laterality occurred in succession.

### MEG data acquisition

All recordings were conducted in a one-layer magnetically shielded room with active shielding engaged for environmental noise compensation. With an acquisition bandwidth of 0.1-330 Hz, neuromagnetic responses were sampled continuously at 1 kHz using an Elekta/MEGIN MEG system (Helsinki, Finland) with 306 sensors, including 204 planar gradiometers and 102 magnetometers. During data acquisition, participants were monitored via real-time audio-visual feeds from inside the shielded room. Each MEG dataset was individually corrected for head motion and subjected to noise reduction using the signal space separation method with a temporal extension [94].

### Structural MRI processing and MEG co-registration

Prior to MEG measurement, four coils were attached to the subject’s head and localized, together with the three fiducial points and scalp surface, with a 3-D digitizer (FASTRAK 3SF0002, Polhemus Navigator Sciences, Colchester, VT, USA). Once the subjects were positioned for MEG recording, an electric current with a unique frequency label (e.g., 322 Hz) was fed to each of the coils. This induced a measurable magnetic field and allowed each coil to be localized in reference to the sensors throughout the recording session. As coil locations were also known with respect to head coordinates, all MEG measurements could be transformed into a common coordinate system. With this coordinate system, each participant’s MEG data were co-registered with their structural T1-weighted MRI prior to source space analysis using BESA MRI (Version 2.0). Structural MRI data were aligned parallel to the anterior and posterior commissures and transformed into standardized space. Following source analysis (i.e., beamforming), each subject’s functional MEG images were also transformed into standardized space using the transform that was previously applied to the structural MRI volume and spatially resampled.

### MEG preprocessing, time-frequency transformation, and sensor-level statistics

Eye blinks and cardiac artifacts were removed from the data using signal space projection (SSP), which was accounted for during source reconstruction [95]. The continuous magnetic time series was divided into epochs of 3500 ms duration, with 0 ms defined as the onset of the cue and the baseline being the -600 to 0 ms window before cue onset. Given our task and epoch design, the target onset occurred at 300 ms. Epochs containing artifacts were removed based on a fixed threshold method, supplemented with visual inspection. In brief, for each individual, the distribution of amplitude and gradient values across all trials were computed, and those trials containing the highest amplitude and/or gradient values relative to the full distribution were rejected by selecting a threshold that excluded extreme values. Importantly, these thresholds were set individually for each participant, as inter-individual differences in variables such as head size and proximity to the sensors strongly affects MEG signal amplitude (average threshold across the sample: 1144.63 femtoTesla (fT), SD = 504.94). On average 86.86 valid and 86.32 invalid trials per participant remained after artifact rejection and were used in subsequent analysis. To ensure there were no systematic differences in the number of trials per participant, an ANCOVA was computed and this showed no significant main effect of condition (F(1,92) = .008, *p* = .930), age (F(1.92) = .236, *p* = .628), or the interaction effect (F(1,92) = .082, *p* = .775).

Artifact-free epochs were transformed into the time-frequency domain using complex demodulation [96], and the resulting spectral power estimations per sensor were averaged over trials to generate time-frequency plots of mean spectral density. These sensor-level data were normalized per time-frequency bin using the respective bin’s baseline power, which was calculated as the mean power during the -600 to 0 ms baseline period. The specific time-frequency windows used for source reconstruction were determined by statistical analysis of the sensor-level spectrograms across all participants and conditions using the entire array of 204 gradiometers. Briefly, each data point in the spectrogram was initially evaluated using a mass univariate approach based on the general linear model. To reduce the risk of false positive results while maintaining reasonable sensitivity, a 2-stage procedure was followed to control for Type-1 error. In the first stage, two-tailed paired-sample t-tests against baseline were conducted on each data point, and the output spectrogram of t-values was thresholded at *p* < 0.05 to define time-frequency bins containing potentially significant oscillatory deviations across all participants. In stage two, time-frequency bins that survived the threshold were clustered with temporally and/or spectrally neighboring bins that were also above the threshold (*p* < 0.05), and a cluster value was derived by summing the t-values of all data points in the cluster. Nonparametric permutation testing was then used to derive a distribution of cluster values, and the significance level of the observed clusters (from stage 1) were tested directly using this distribution [97, 98]. For each comparison, at least 10,000 permutations were computed. Based on these analyses, the time-frequency windows that contained significant oscillatory events across all participants and conditions were subjected to the beamforming analysis. Further details about our MEG data processing pipeline are available in a recent publication [99].

### MEG source imaging and statistics

Cortical networks were imaged using the dynamic imaging of coherent sources (DICS) beamformer [100], which applies spatial filters in the time-frequency domain to calculate voxel-wise source power for the entire brain volume. The single images were derived from the cross-spectral densities of all combinations of MEG gradiometers averaged over the time-frequency range of interest, and the solution of the forward problem for each location on a grid specified by input voxel space. Following convention, we computed noise-normalized source power for each voxel per participant using active (i.e., task) and passive (i.e., baseline) periods of equal duration and bandwidth [101] at a resolution of 4.0 x 4.0 x 4.0 mm. Such images are typically referred to as pseudo-t maps, with units (pseudo-t) that reflect noise-normalized power differences (i.e., active versus passive) per voxel. MEG pre-processing and imaging used the Brain Electrical Source Analysis (version 6.1) software.

After imaging, average whole-brain maps were computed across all conditions (valid and invalid) and participants for the selected time-frequency bands. These 3D maps of brain activity were used to assess the anatomical basis of the significant oscillatory responses identified through the sensor-level analysis. Conditional whole-brain images per time-frequency response were then subtracted (i.e., invalid – valid) within each participant to generate maps representing the validity effect (i.e., attention reallocation). To assess the impact of chronological age on validity-related neural oscillations, these validity maps were subjected to whole-brain voxel-wise correlation analyses, with age as the covariate of interest. To control for Type-I error, maps were thresholded at *p* < 0.01 to define potentially significant clusters and then nonparametric permutation testing was conducted, similar to that performed on the sensor-level spectrograms, with at least 10,000 permutations per comparison. Finally, a mediation analysis using regression was conducted [45], followed by bootstrapping [46], for added rigor (SPSS Version 23.0, IBM Analytics, Amonk, New York, USA).

### Data availability

The data that support the findings of this study are available from the corresponding author, Dr. Tony W. Wilson, upon reasonable request.
